# Exploring the Potential of Flour and Protein Concentrate From Richlea Lentils in the Development of Couscous With a Pilot‐Scale Twin‐Screw Extruder

**DOI:** 10.1111/1750-3841.70356

**Published:** 2025-07-14

**Authors:** Edwin Allan, Dilpreet Bajwa, Girish M. Ganjyal, Chidimma Ifeh, Ravi Pirati, Wan‐Yuan Kuo

**Affiliations:** ^1^ Sustainable Food Systems Program, Department of Food Systems, Nutrition and Kinesiology Montana State University Bozeman Montana USA; ^2^ Mechanical Engineering Program, Department of Mechanical and Industrial Engineering Montana State University Bozeman Montana USA; ^3^ Food Processing Extension and Research Program, School of Food Science Washington State University Pullman Washington USA; ^4^ Chemical Engineering, Department of Chemical and Biological Engineering Montana State University Bozeman Montana USA

## Abstract

Lentil couscous was developed with different ratios of lentil protein concentrate to lentil flour and different extruder screw speeds using a twin‐screw pilot scale extruder at a dry feed rate of 10 kg/h and a water feed rate of 3 kg/h. An analysis of the bulk density, the water absorption index, instrumental texture (hardness, springiness, cohesiveness, gumminess, chewiness, and resilience) was conducted for all 12 formulations. Five lentil couscous formulations were then presented to 120 participants to evaluate sensory properties (sensory acceptance in terms of overall liking, appearance liking, flavor liking, and texture liking, as well as check all that apply) and purchasing indicators (purchase intent and willingness to pay). An increase in the ratio of lentil protein concentrate to lentil flour from 0:100 to 40:60, decreased the water absorption index from 5.58 to 4.29, and consequentially decreased the springiness, cohesiveness, chewiness, and resilience texture. Higher overall liking, appearance liking, flavor liking, and texture liking for couscous presented to participants were observed for the formulations with lentil protein concentrate levels at 20% or less and a screw speed of 250–300 rpm. Check all that apply tests revealed nutty and a nutty aftertaste positively impacted flavor and overall liking (*p* < 0.05) while grainy texture attribute negatively impacted the texture and overall liking (*p* < 0.05).

## 1 Introduction

Couscous is a popular food in Northern Africa, some Sub‐Saharan African countries, and recently North America (Igrejas et al. 2020; Moussa et al. 2022; Hammami et al. 2022). Couscous is a granular product of 1–2 mm in diameter (Chemache et al. 2021; Moussa et al. 2022), typically made from durum wheat but has also been made with sorghum, millet, or corn in West Africa (Aboubacar and Hamaker 1999). Couscous is considered a healthy food, ideal for vegetarians (Coskun 2013) and provides complex carbohydrates, B vitamins, and minerals (Çelik et al. 2004). Once mostly in the healthy food sections of grocery stores in North America (Coskun 2013), the increased consumption of couscous has widened its use in other recipes like salads (Yüksel et al. 2018) and breakfast cereals (Aboubacar and Hamaker 1999) and therefore has inspired the demand for gluten‐free options. Evidently, one‐third of adult Americans have reported wanting to reduce or avoid gluten consumption (NPD Group 2015), reflecting the $4.6 billion in sales of gluten‐free products in 2017 (Marketsandmarkets.com 2018). With the United States ranking number four in couscous imports (Yüksel et al. 2018), attempts are being made to develop gluten‐free couscous (Aboubacar and Hamaker 1999; Demir and Demir 2016). Supporting information

Lentil flour is gluten‐free and contains higher amounts of protein, fiber, and phytochemicals than traditional cereal flours such as wheat, rice, and corn (Bresciani et al. 2021; Romano et al. 2021), making it a viable candidate for making nutritionally enhanced couscous. Traditional couscous is prepared by mixing and aggregating flour with water into small granules, steaming, and then drying (Moussa et al. 2022). The gluten in wheat couscous forms a strong viscoelastic network, creating optimal dough properties which, after hydration, present favorable culinary qualities such as stability, higher weight per volume, lower hydration times, and lower loss of solids (da Silva et al. 2016). Creating more nutritious gluten‐free couscous has been a challenge due to the lowered structural integrity caused by the absence of gluten (Benayad et al. 2021). One study showed a decreased textural stability in cooked couscous when replacing more than 25% durum wheat flour with lentil flour (Benayad et al. 2021; Demir and Demir 2016). Gluten‐free pasta, and therefore couscous, made from lentil flour can present desirable culinary properties (Bresciani et al. 2021) if a stronger texture is created from new mechanized methods such as extrusion (Romano et al. 2021).

Extrusion processing generates elevated temperature and pressure which is valuable for processing gluten‐free ingredients (Pasqualone et al. 2020). Extrusion is performed with a single or twin‐screw extruder, which partially or fully cooks food ingredients in a chamber called the barrel and then shapes and pushes the cooked material out of a hole called a die (Berrios et al. 2022; Pasqualone et al. 2020). The rotation of the extruder screw against the food material generates temperature and pressure through friction, which increase with higher screw speed (Berrios et al. 2022). Extrusion processing uses elevated temperature and pressure to pregelatinize and gelatinize starch and cross‐link proteins to create a strong dough matrix (Ek et al. 2021; Riaz 2011). The gluten‐free products created from extrusion therefore have a uniform and cohesive texture to withstand additional cooking processes (Bresciani et al. 2021). Gluten‐free pearl millet couscous developed from an extrusion study was observed to have a comparable overall liking to traditional durum wheat couscous (Moussa et al. 2022).

Extrusion also presents the opportunity to increase the nutrition content of couscous by including high fiber or protein found to be lacking in some gluten‐free products (Woomer and Adedeji 2021). The recent production of protein concentrates from lentil flour using mechanical separation methods (González and Pérez 2002) enables the innovation of creating a 100% lentil couscous with increased protein content using extrusion. Consequently, the development of lentil couscous with desired structural integrity addresses the consumer appreciation for whole lentil products, and the desire for a “clean label” in the United States (Milner et al. 2020). Therefore, the objective of this study was to examine the use of a twin‐screw extruder in developing a 100% lentil couscous and determine the effect of increasing protein and screw speed on the quality, texture, and sensory properties of the lentil couscous.

## 2 Materials and Methods

### 2.1 Materials

Organic lentil flour and lentil protein concentrate from the variety CDC Richlea was obtained from Montana Pure Protein (Billings, MT, USA). As per information from the vendor, lentil protein concentrate was produced from the air classification (Assatory et al. 2019) of lentil flour. Gold Medal Semolina No. 1 (53162000) was purchased from General Mills (Great Falls, MT, USA).

#### 2.1.1 Proximate Analysis

Lentil flour and lentil protein concentrate samples were analyzed by NP Analytical Laboratories (St. Louis, MO, USA) for ash, crude fiber, crude protein, starch, and moisture, using the Association of Official Analytical Collaboration (AOAC) Methods 942.05, 962.09, 990.03, 996.11, and 930.15, respectively.

#### 2.1.2 Central Composite Design for Lentil Couscous Formulations

Preliminary trials were conducted to choose suitable ranges of formulations and extrusion parameters for making lentil couscous. Based on the preliminary trials, the ratio of lentil flour to lentil protein concentrate and the extruder screw speed were critical determinants of the product's cooking quality, flavor, and texture. A central composite design was conducted with RStudio Version 1.2.1335 (RStudio Inc., Boston, MA, USA) to evaluate the effects of two formulation factors on the bulk density, water absorption index (WAI), instrumental texture, and sensory properties of the lentil couscous. Factor I represents the weight ratio of lentil protein concentrate to lentil flour (d.b.) and factor II represents the extruder screw speed (Table 1). The corresponding values for the two factors with three levels of variation (2^3^, *α* = 2) and their combinations for each formulation were determined using a central composite design (Table 1). The response variables were analyzed and modeled using the equation:

$$Y=\beta_{0}+\Sigma \beta_{i}X_{i}+\Sigma \beta_{ii}X_{i}^{2}+\Sigma \beta_{ij}X_{i}X_{j}+\varepsilon$$

where Y is the predicted response; $\beta_{0}$ is a constant; $\beta_{i}$ is the linear coefficient; $\beta_{ii}$ is the squared coefficient; $\beta_{ij}$ is the cross‐product coefficient, and ε is the random error associated with the response. Three‐dimensional response surfaces were made for each response variable using SAS 9.4 (Cary, NC, USA) according to the predicted model equation.

**TABLE 1:** Coded levels and corresponding values for the lentil couscous formulations generated from a central composite design.

Formulations	Block	Factor I		Factor II		Nutrient content (g) per 140 g of input materials (w.b.)			
		Coded level (*x*_1_)	Weight ratio of lentil protein concentrate to lentil flour (d.b.)	Coded level (*x*_2_)	Extruder screw speed/(rpm)	Starch	Protein	Fiber	Ash
1‐30/70/225	1	1	30:70	−1	225	65.1	41.0	10.4	5.2
2‐30/70/275	1	1	30:70	1	275	65.1	41.0	10.4	5.2
3‐10/90/225	1	−1	10:90	−1	225	74.9	33.9	8.6	4.3
4‐10/90/275	1	−1	10:90	1	275	74.9	33.9	8.6	4.3
5‐20/80/250	1	0	20:80	0	250	70.0	37.5	9.5	4.7
6‐20/80/250	1	0	20:80	0	250	70.0	37.5	9.5	4.7
7‐0/100/250	2	−2	0:100	0	250	79.8	30.4	7.7	3.8
8‐40/60/250	2	2	40:60	0	250	60.2	44.5	11.4	5.6
9‐20/80/300	2	0	20:80	2	300	70.0	37.5	9.5	4.7
10‐20/80/200	2	0	20:80	−2	200	70.0	37.5	9.5	4.7
11‐20/80/250	2	0	20:80	0	250	70.0	37.5	9.5	4.7

#### 2.1.3 Extrusion Cooking

**FIGURE 1:**
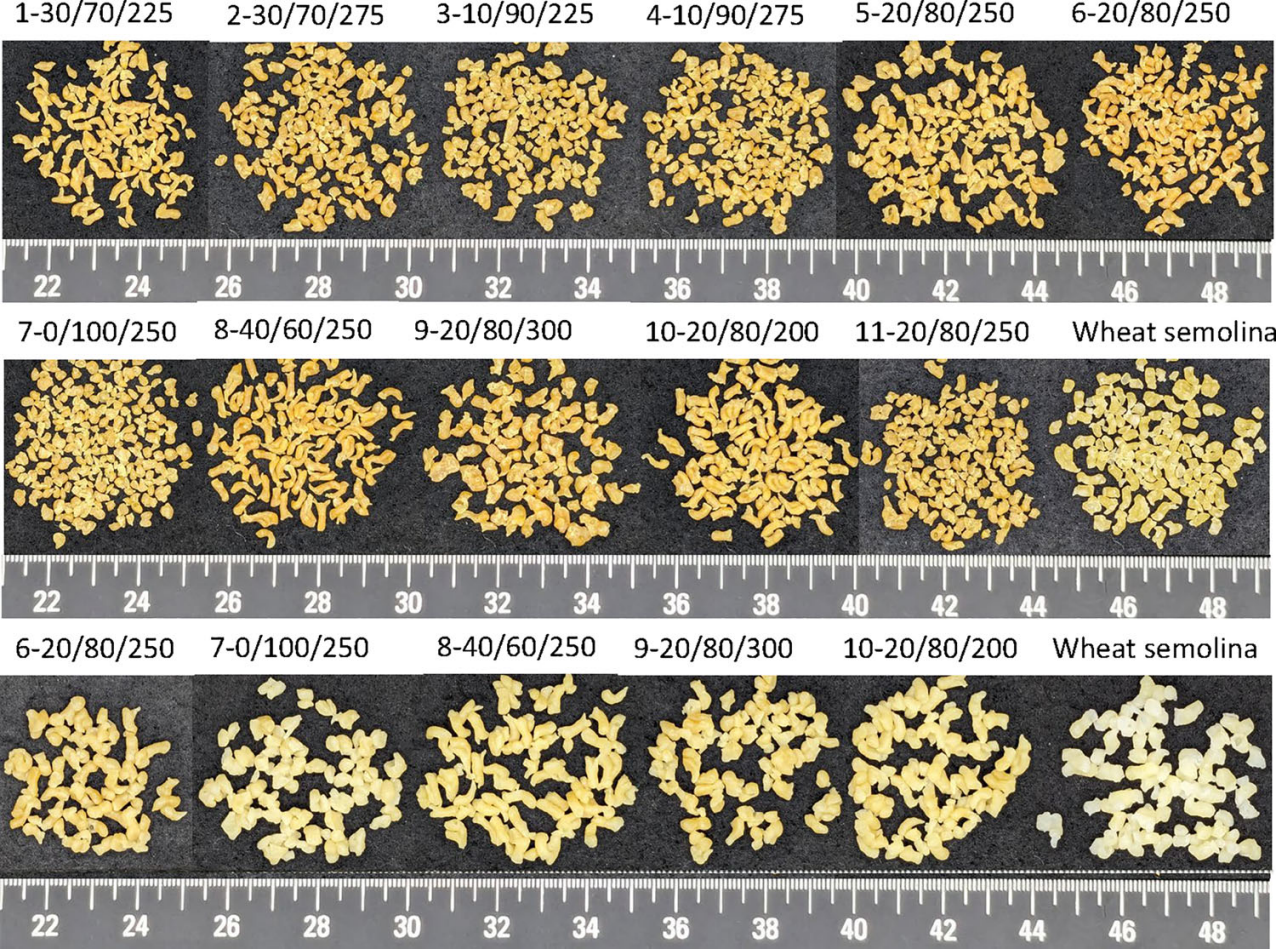
Lentil couscous formulations showing formulation 1 on the first row to formulation 11 on the second row. Hydrated lentil couscous formulations used for the sensory consumer acceptance test on the third row. The last cell on the second and third row shows the reference wheat semolina.

Extrusion cooking was performed with a pilot scale corotating twin‐screw extruder (BCTM‐30, Buhler Inc., St. Paul, MN, USA) with a screw diameter (*D*) of 30 mm and a 20*D* process length. The extruder barrel had four heating zones and a feeding zone. A dry feed rate of 10 kg/h (d.b.), a feed moisture level of 2 kg/h and varying screw speeds (Table 1) were used to prepare lentil couscous. A 20‐hole circular extruder die with a diameter of 2.0 mm per hole was used to shape the couscous into grain‐like spheres (Figure 1). A variable speed die face cutter with three blades moving at 250 rpm was used to cut couscous into an approximately 0.5 mm length (Figure 1). The control wheat semolina couscous was however extruded at a dry feed rate of 6 kg/h (d.b.), a feed moisture level of 2.5 kg/h and a lower screw speed of 150 rpm. Couscous was collected when the system achieved stability, characterized by temperatures of 105°C at the die with stable specific mechanical energy and backpressure at the die. Temperatures at the heating zones were also measured at 119°C, 139°C, 125°C, and 53°C at zones 4, 3, 2, and 1, respectively. The couscous was then dried in a proofing oven (Metro C5 3 Series Insulated Holding/Proofing Cabinet, InterMetro Industries Corp, USA) at 40°C for 18 h to a moisture content of 5.1 ± 0.2% (w.b.). The dried couscous was stored in air‐tight plastic bags at room temperature for subsequent analyses.

#### 2.1.4 Physical Analyses

##### 2.1.4.1 Bulk Density

Bulk density was evaluated according to Kaur et al. (2013). The samples were gently filled in a 10 mL graduated cylinder, previously tared. The bottom of the cylinder was gently tapped on a laboratory bench several times until there was no further diminution of the sample level after filling to the 10 mL mark. Then, bulk density in g/cm^3^ was calculated according to the equation below.

(1) $$\mathrm{Bulk}\mathrm{density}=\frac{a}{b}$$

where *a* is the mass of sample in grams and *b* is the volume of sample in cm^3^.

##### 2.1.4.2 Water Absorption Index

The WAI was determined according to Anderson et al. (1970) with modifications. A 1‐g sample was dispersed in 10 g of distilled water at 100°C, using a glass rod to disaggregate any lumps. After 30 min at room temperature, the mixture was rinsed into tared centrifuge tubes, made up to 32.5 g and centrifuged at 3000 × *g* for 10 min. Then, the sediment was weighed for its solid content and was used to calculate the WAI according to the equation below:

(2) $$\mathrm{WAI}=\frac{c}{d}$$

where *c* is the weight of sediment and *d* is the weight of dry solid.

##### 2.1.4.3 Texture Analysis

Texture profile analysis was performed with the TA.XT Plus C texture analyzer and software (Texture Technologies, La Crescenta, CA, USA). The texture analyzer was equipped with a TA‐25c 4‐inch diameter cylindrical probe and a 50 kg load cell. The couscous was rehydrated in 95°C water a 1:1.5 couscous/water ratio (w/w) and left to soak for 30 min at room temperature with intermittent stirring. The couscous was placed in a fitting cylinder and underwent a compression at 1.0 mm/s at room temperature until reaching 50% strain, after which the probe was pulled away from the sample and repeated another compression at the same speed and strain. The parameters obtained from TPA included hardness (peak force after the first compression, N), cohesiveness (area of work, Ns during the second compression divided by the area of work, Ns during the first compression), springiness (distance of the detected height during the second compression divided by the original compression distance), gumminess (hardness × cohesiveness), chewiness (gumminess × springiness), and resilience (upstroke energy of the first compression divided by the downstroke energy of the first compression). These parameters were calculated based on the force of compression and the height of compressed samples (Texture Technologies Corp. 2020). For each couscous formulation, five measurements were taken.

#### 2.1.5 Sensory Evaluations

Approval (2023‐881‐EXEMPT) from the Institutional Review Board at Montana State University was received before the sensory evaluations were carried out. The sensory evaluations were performed according to Benayad et al. (2021). Five lentil couscous formulations (6, 7, 8, 9, and 10) were selected to be evaluated against wheat semolina to prevent sensory consumer fatigue. Formulations 6, 9, and 10 represent the midpoint of the ratio of lentil protein concentrate to lentil flour (20/80) with varying levels of extruder screw speed (200, 250, and 300). Formulations 7 and 8 represent the midpoint of extruder screw speed (250 rpm) with extreme levels of the ratio of lentil protein concentrate to lentil flour (0/100 and 40/60). For sensory evaluation, couscous samples were hydrated for 15 min at a 1:1.5 ratio (w/w) in an aqueous mixture at an initial temperature of 95°C but included 0.6% (w/w) olive oil and 0.01% (w/w) table salt so samples could be presented as a couscous dish (Çelik et al. 2004).

The sensory evaluations were performed with 108 participants in a room with restaurant‐style tables at room temperature. Redjade (Martinez, CA, USA) was used to code samples with three‐digit random codes and completely randomize the order of sample presentation across participants. Participants were instructed to cleanse their palates using room‐temperature water before tasting the next sample. The participants rated overall appearance, flavor, and texture likings on a nine‐point hedonic scale from “Dislike extremely” (1), “Neither like nor dislike” (5) to “Like extremely” (9). A check all that apply (CATA) test was also conducted for each sample, following the hedonic scales. Participants were asked to check from a list of sensory terms they considered appropriate to describe the samples' appearance, flavor, texture, and aftertaste (Chávez et al. 2021). The list of CATA terms was generated from a preliminary test with a panel of three certified executive chefs.

Purchase intent (PI) and willingness to pay (WTP) for Formulation 6 were also evaluated according to Garg et al. (2023). The participants were first presented with the local market price of wheat semolina couscous ($4.71 per 10 oz). For PI, they were asked if lentil couscous becomes available in Montana, how likely are they to buy it if it is $4.71 per 10 oz (dried weight) at a grocery store? The five‐point scale was anchored from “Definitely will not buy” (1), “Might or might not buy” (3), and “Definitely will buy (5)” (Delgado et al. 2013). For WTP, they were asked how much they were willing to pay for 10 oz (dried weight) of this lentil couscous, on a five‐point scale anchored between $1.71 and $5.71, with incremental intervals of $1. Participants’ demographic information (gender, age range, ethnicity, and frequency of lentil and couscous consumption) was also collected.

#### 2.1.6 Statistical Analyses

The data from physical analyses, sensory liking, PI, and WTP in this study were processed using R Version 1.2.1335 (R Core Team., Boston, MA, USA). A multilinear regression model was fit to determine the effects of the ratio of lentil protein concentrate to lentil flour and extruder screw speed on the bulk density, WAI and instrumental texture of lentil couscous formulations. We applied a one‐way analysis of variance (ANOVA) to determine the differences in means among the lentil couscous and the reference wheat semolina couscous. A pairwise comparison between groups was conducted using Fisher's least significant difference test (*α* = 0.05).

Data from demographic information and CATA tests were analyzed using XLSTAT 2020.1 (Addinsoft Inc, New York, NY, USA). For CATA, Cochran's *Q* test and multiple pairwise comparisons with Sheskin's critical difference were conducted to compare differences in the attribute selection frequencies among the samples. Agglomerative hierarchical clustering was used to identify clusters among the panelists using overall liking data. A Fisher's exact test was used to detect noticeable differences in the participants’ demographic breakdown in each cluster, compared to the pooled population (*α* = 0.05).

Means, standard deviations, and Pearson's correlation coefficients for the relationships between all properties were calculated using XLSTAT (XLSTAT 2020.1, Addinsoft Inc, New York, NY, USA). Principal coordinate analysis was used to visualize the relationships between checked attributes and overall liking, flavor liking and texture liking.

## 3 Results

### 3.1 Proximate Analyses

The organic lentil flour was comprised 2.72% ash, 1.84% fat, 5.52% fiber, 21.70% protein, 57.00% starch, and 8.21% moisture (w/w, d.b.); and the lentil protein concentrate, 6.00% ash, 3.90% fat, 12.00% fiber, 47.00% protein, 47.00% starch, and 7.55% moisture.

### 3.2 Bulk Density

The bulk density of our extruded lentil couscous formulations presented a range from 0.82 ± 0.14 g/cm^3^ to 1.12 ± 0.13 g/cm^3^ (Table 2). The bulk density of wheat semolina couscous produced using the traditional steaming method ranged from 0.65 to 0.67 (Boudouira et al. 2023) with the incorporation of lentil flour at 30% presenting no effect. An increase in bulk density was, however, observed by Benayad et al. (2021) with ≥50% incorporation of lentil flour. No differences in bulk density were observed between our lentil couscous formulations 3–11 (*p* = 0.09, Table 2). Comparing the lentil couscous formulations 1–3 and 2–4, at the same screw speed, formulations 1 and 2 with a higher level of lentil protein concentrate (30%, d.b., in the dry feed) had noticeably lower bulk densities than their counterparts, formulations 3 and 4, with a 10% inclusion of lentil protein concentrate (*p* < 0.05, Table 2). Protein functionality is heavily dependent on solubility, with lower protein molecular weight being associated with greater solubility (Lopes‐da‐Silva and Monteiro 2019). Lentils are recognized for their high protein solubility (Jarpa‐Parra 2018) which in relatively higher moisture (> 15%) extrusion conditions compared to extruded snacks, results in reduced aggregation (Lopes‐da‐Silva and Monteiro 2019), and a lower overall bulk density.

**TABLE 2:** Bulk density and hydration properties of lentil couscous formulations compared to the reference product.

Formulations	Bulk density (g/cm^3^)	Water absorption index (WAI)
1‐30/70/225	0.91 ± 0.09^bc^	5.09 ± 0.20^ab^
2‐30/70/275	0.82 ± 0.14^c^	4.68 ± 0.66^ab^
3‐10/90/225	1.12 ± 0.13^a^	5.37 ± 0.69^a^
4‐10/90/275	1.07 ± 0.00^ab^	5.24 ± 0.14^a^
5‐20/80/250	1.11 ± 0.07^a^	5.37 ± 0.12^a^
6‐20/80/250	1.00 ± 0.06^abc^	5.10 ± 0.40^ab^
7‐0/100/250	1.00 ± 0.06^abc^	5.58 ± 0.53^a^
8‐40/60/250	0.96 ± 0.21^abc^	4.29 ± 0.87^b^
9‐20/80/300	0.97 ± 0.00^abc^	5.02 ± 1.05^ab^
10‐20/80/200	0.95 ± 0.13^abc^	4.80 ± 0.33^ab^
11‐20/80/250	0.95 ± 0.13^abc^	5.36 ± 0.06^a^
Wheat semolina	1.04 ± 0.06^ab^	5.36 ± 0.06^a^

For the same column, values (means ± SD) followed by the same letter are not significantly different based on Fisher's least significant difference test (*α* = 0.05).

**FIGURE 2:**
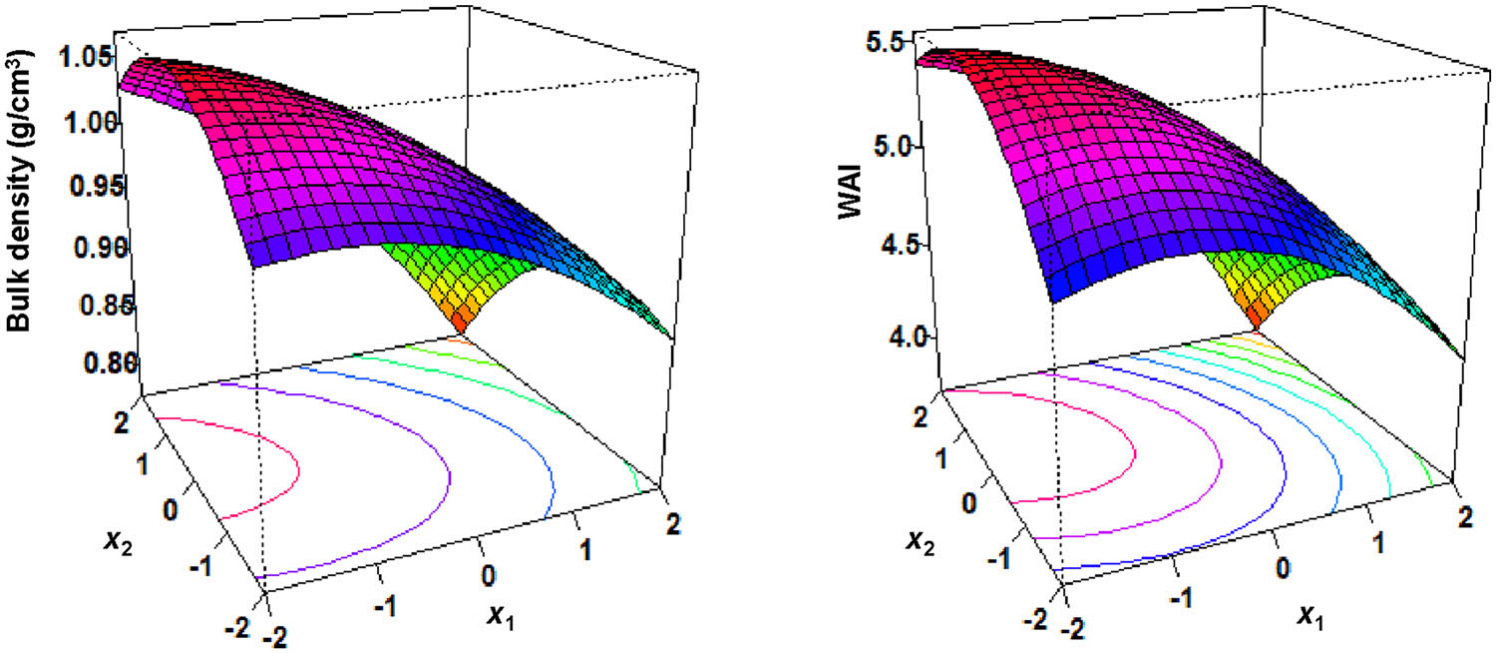
Response surfaces showing the bulk density and water absorption index (WAI), WAI of lentil couscous formulations. *X*_1_ and *X*_2_ are the RSM‐coded levels for the weight ratio of lentil protein concentrate to lentil flour and extruder screw speed, respectively (Table 1).

In general, higher levels of protein in extrusion result in a denser product, while an increase in screw speed results in higher temperatures which increase gelatinization and reduce bulk density (Allen et al. 2007). Factors *x*_1_ and *x*_2_, however, had no noticeable influence on bulk density (Figure 2 and Table 4). The unexpected results may again be attributed to the high protein solubility of lentil protein ranging from 78% to 91% (Can Karaca et al. 2011; Joshi et al. 2011) which limits the protein's ability to restrict the expansion during extrusion. This phenomenon was especially influential during our couscous extrusion which utilized a water rate of 2 kg/h, 6% higher than the typical water rate used in the extrusion of expanded products which typically show more variation in bulk density (Ek et al. 2021). Weak evidence (*p* = 0.097) of differences observed between lentil couscous formulations in Table 2 can be attributed to the high protein solubility of lentil protein concentrate. Furthermore, all couscous formulations were extruded to a minimum of 105°C at the die with ∼17% moisture more than sufficient for starch gelatinization and swelling (Ek et al. 2021), further explaining the absence of a relationship between bulk density and factor *x*_2_.

Remarkably, the bulk density of the wheat semolina couscous was not different from the lentil couscous formulations despite being extruded at a lower screw speed of 150 rpm. This can be attributed to the action of gluten in wheat semolina which creates a strong viscoelastic network and cohesive forces leading to a compact dense structure (Benayad et al. 2021; Boudouira et al. 2023). Formulation 2, with a screw speed of 275 rpm, however, had a noticeably lower bulk density (*p* < 0.05, Table 2) compared to wheat semolina couscous. The comparable bulk densities between wheat semolina couscous and most of the lentil couscous formulations suggest that the handling, packaging, transport, and storage of commercial wheat semolina couscous (Benayad et al. 2021) can be applied to most of the lentil couscous formulations (1, 3, and 4–11).

### 3.3 Water Absorption Index

The WAI provides information on the amount of water a sample can absorb (Giovanelli et al. 2023) and is a notable property of couscous (Ren et al. 2021). The WAI of lentil couscous formulations ranged from 4.29 ± 0.87 to 5.58 ± 0.53 (Table 2) similar to the range (3.7–5.07) observed for the WAI of extruded semolina couscous (Debbouz and Donnelly 1996).

Formulation 8 with 40% lentil protein concentrate had a noticeably lower WAI compared to formulations 3, 4, 5, 7, and 11 (*p* < 0.05). Protein content above 15% in couscous formulations was reported by Debbouz (1992) to noticeably lower the WAI. Consistent with this literature, *x*_1_ was negatively correlated with the WAI (Table 4). Starch polymers are hydrophilic and therefore in extrusion processing, starch granules swell after absorbing a minimum of 20% w/w moisture with their free hydroxyl groups (Heydari et al. 2022; Shang et al. 2008). The high moisture (>20% w/w) and temperature (>60°C) during the extrusion would create pregelatinized starch in the extruded and dried couscous which can readily absorb water when rehydrated (Giovanelli et al. 2023). The increase in lentil protein concentrate levels (*x*_1_) lowers starch contents in couscous formulations (Table 1), thus reducing the concentration of free hydroxyl groups for moisture absorption when rehydrated.

The WAIs of all lentil couscous formulations, excluding formulation 8 (*p* value = 0.08), were not noticeably different from the wheat semolina couscous (Table 2). Our findings contradict Benayad et al. (2021) who reported lentil couscous (unextruded) having higher water absorption properties compared to wheat semolina couscous due to their higher proteins and fibers. We attribute the lower WAI of our lentil couscous compared to wheat semolina to the higher temperatures (≥105°C) achieved during extrusion, which cause proteins to cross‐link and become compact, limiting water absorption (Kim 2018). In addition, the higher water absorption could be associated with compositional and physicochemical characteristics of lentil flour and lentil protein concentrate ingredients such as particle size and pH which contribute to final product characteristics (Farooq and Boye 2011). Nevertheless, the WAI of wheat semolina couscous typically presents a desirable swelling characteristic (Benayad et al. 2021; Hammami et al. 2022). Thus, the comparable WAI between most of the lentil couscous formulations and the wheat semolina couscous suggests a promising culinary property and market potential of the lentil couscous.

### 3.4 Instrumental Texture

**FIGURE 3:**
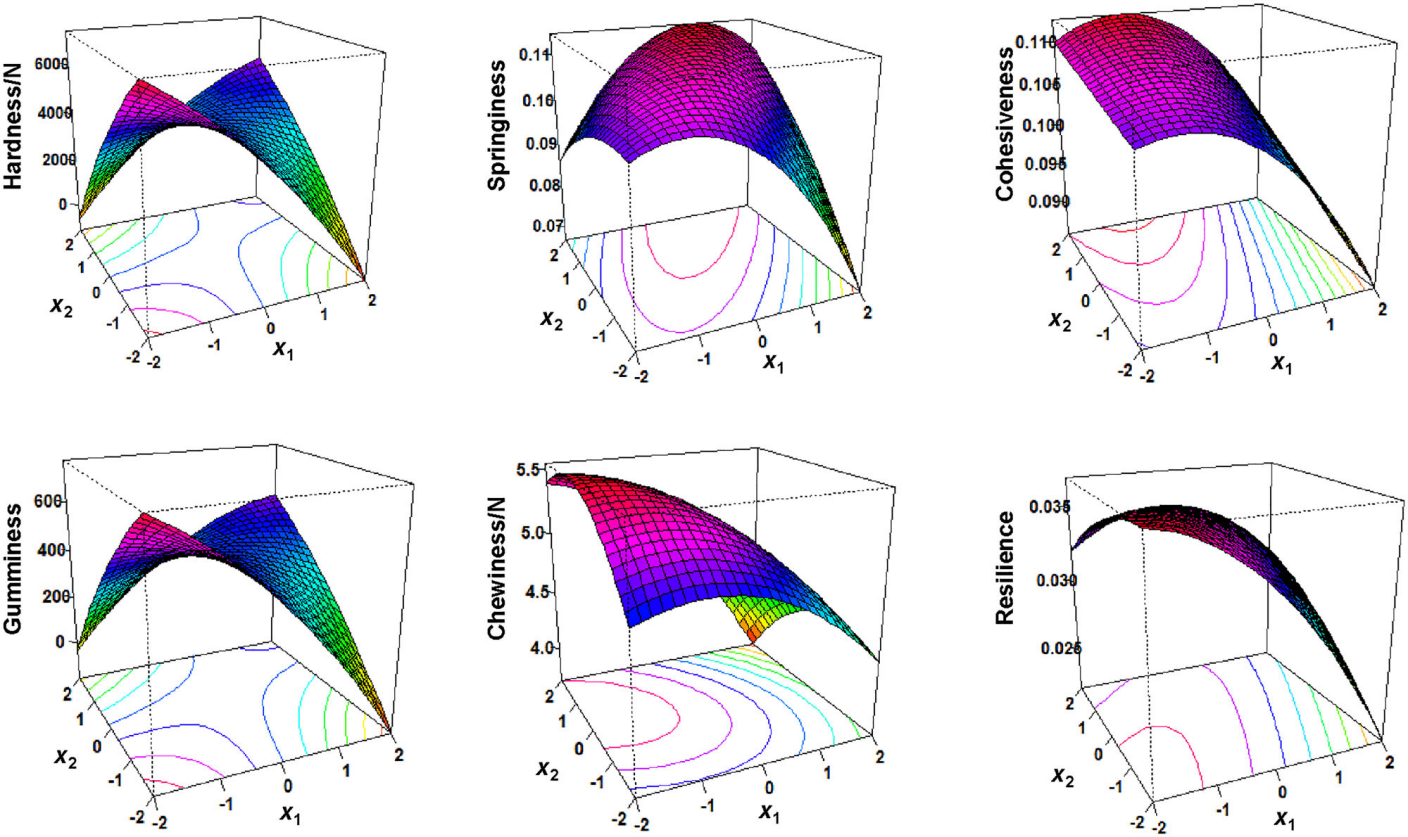
Response surfaces showing the instrumental texture, hardness, springiness, cohesiveness, gumminess, chewiness, and resilience of lentil couscous formulations. *X*_1_ and *X*_2_ are the RSM‐coded levels for the weight ratio of lentil protein concentrate to lentil flour and extruder screw speed, respectively (Table 1).

Couscous texture should not be too hard/firm and easy to chew after hydration (Hammami et al. 2022). Extrusion conditions including screw speed can affect product hardness with Moussa et al. (2022) reporting lower hardness of extruded millet couscous compared to a traditionally produced millet couscous control. In general, the hardness of lentil couscous decreased with an increase in *x*_1_ and *x*_2_, similar to gumminess (Figure 3 and Table 3). Gumminess is derived from the product of hardness and cohesiveness measurements (Texture Technologies Corp. 2020) explaining the similar trends between hardness and gumminess. Cohesiveness, however, typically has subtle differences for soft, hydrated products (Yu et al. 2018), and, therefore, unlike hardness, cohesiveness did not significantly impact gumminess.

**TABLE 3:** Instrumental texture of lentil couscous formulations compared to the reference product.

Formulations	Hardness (N)	Springiness	Cohesiveness	Gumminess	Chewiness (N)	Resilience
1‐30/70/225	3841.0 ± 147.2^de^	0.09 ± 0.01^c^	0.10 ± 0.00^fg^	366.0 ± 3.8^d^	33.84 ± 1.89^gh^	0.0323 ± 0.0015^ef^
2‐30/70/275	5891.2 ± 680.2^b^	0.11 ± 0.01^ab^	0.10 ± 0.00^ef^	599.5 ± 77.8^b^	63.82 ± 8.49^b^	0.0343 ± 0.0012^cde^
3‐10/90/225	6739.7 ± 473.8^a^	0.10 ± 0.00^bc^	0.11 ± 0.01^cde^	708.4 ± 11.3^a^	71.89 ± 1.99^a^	0.0373 ± 0.0012^b^
4‐10/90/275	5157.8 ± 83.1^c^	0.10 ± 0.00^bc^	0.11 ± 0.00^bcd^	565.3 ± 16.1^b^	57.26 ± 0.34^c^	0.036 ± 0.001^bc^
5‐20/80/250	3686.7 ± 185.5^de^	0.12 ± 0.01^a^	0.11 ± 0.00^bc^	411.9 ± 18.0^cd^	48.15 ± 3.10^d^	0.034 ± 0.001^cde^
6‐20/80/250	1907.2 ± 206.4^h^	0.12 ± 0.00^a^	0.11 ± 0.00^bcd^	211.1 ± 18.8^f^	24.38 ± 1.97^ij^	0.034 ± 0.0017^cde^
7‐0/100/250	2914.3 ± 27.7^g^	0.10 ± 0.01^bc^	0.11 ± 0.00^bcd^	316.7 ± 9.0^e^	32.25 ± 1.54^gh^	0.0353 ± 0.0006^bcd^
8‐40/60/250	2214.4 ± 211.5^h^	0.09 ± 0.00^c^	0.09 ± 0.00^g^	207.8 ± 21.6^f^	19.62 ± 2.63^j^	0.0263 ± 0.0006^g^
9‐20/80/300	2256.4 ± 224.0^h^	0.12 ± 0.01^a^	0.11 ± 0.00^b^	252.4 ± 23.4^f^	28.86 ± 3.12^hi^	0.034 ± 0.001^cde^
10‐20/80/200	3473.1 ± 92.7^ef^	0.10 ± 0.00^bc^	0.11 ± 0.00^de^	365.6 ± 9.2^d^	37.41 ± 1.95^fg^	0.0317 ± 0.0006^f^
11‐20/80/250	4000.8 ± 163.5^d^	0.11 ± 0.01^ab^	0.11 ± 0.01^de^	418.6 ± 7.0^c^	46.18 ± 2.77^de^	0.0333 ± 0.0006^def^
Wheat semolina	3105.0 ± 225.2^fg^	0.11 ± 0.01^ab^	0.12 ± 0.01^a^	383.9 ± 39.7^cd^	42.41 ± 3.42^ef^	0.0433 ± 0.0025^a^

For the same column, values (means ± SD) followed by the same letter are not significantly different based on Fisher's least significant difference test (*α* = 0.05).

For lentil couscous formulations 1 and 2 (30% protein concentrate for *x*_1_), hardness and gumminess increased with an increase in *x*_2_ (Table 3). Conversely, for formulations 3 and 4 (10% protein concentrate), hardness and gumminess decreased with an increase in *x*_2_. At higher protein concentrations, higher screw speeds generate higher temperatures which cause proteins to denature, cross‐link, and rearrange forming firm and compact structures. However, at higher starch concentrations, an increase in screw speed causes an increase in gelatinization resulting in softer and more cohesive couscous. This phenomenon continues in formulations 5, 6, 9, 10, and 11 (20% protein concentrate) which showed a decrease in hardness and gumminess in moving from screw speeds of 200–300 rpm. No differences (*p* > 0.05) in hardness and gumminess were observed in moving from screw speeds of 200–250 rpm and 250–300 rpm. For formulations 7 and 8 with screw speeds of 250 rpm, hardness and gumminess decreased with an increase in *x*_1_.

Despite the trends observed in the measured hardness and gumminess (Table 3), our linear model showed that neither hardness nor gumminess was influenced by *x*_1_ or *x*_2_ (Table 4). Similar results were observed by Boudouira et al. (2023) with the addition of pulse flours up to 30% having no effect on hardness. Here again, the unexpected results may be attributed to the high protein solubility of lentil protein (Can Karaca et al. 2011; Joshi et al. 2011), which increases the solubility of hydrated couscous, especially in formulations with lower screw speed and lower protein concentrations, unfavorable for protein cross‐linking. Higher levels for *x*_1_ (beyond 40% lentil protein concentrate) may be needed to understand the effect of the selected formulation factors on hardness and gumminess.

**TABLE 4:** Regression coefficients for the determination of the physical properties of lentil puff formulations.

Parameters^a^	Bulk density (g/cm^3^)^b^	Water absorption index^b^	Hardness (N)^b^	Springiness^b^	Cohesiveness^b^	Gumminess^b^	Chewiness (N)^b^	Resilience^b^
bIntercept	1.016***	5.275***	4230.24**	0.111***	0.10735***	449.663**	5.27***	0.0347***
*x*_1_	−0.044	−0.284**	−297.09	−0.002	−0.00400*	−43.85	−0.28**	−0.0021**
*x*_2_	−0.009	−0.008	−163.76	0.003	0.00206	−11.31	−0.01	0.0004
*x*_1_:*x*_2_	−0.010	−0.069	908.01	0.004	0.00050	94.16	−0.07	−0.0008
*x*_1_^2^	−0.009	−0.085	−222.96	−0.004*	−0.00186	−27.64	−0.09	−0.0008
*x*_2_^2^	−0.015	−0.092	−147.87	−0.001	−0.00002	−15.95	−0.09	0.0003

^a^ *x*_1_ is the coded level for the weight ratio of lentil protein concentrate to lentil flour; *x*_2_ is the coded level for extruder screw speed; *x*_1_:*x*_2_ is the interaction of factors 1 and 2; *x*_1_^2^ is the second order of factor 1; *x*_2_^2^ is the second order of factor 2.

^b^ Values superscripted with***, **, and * indicated that the regression provided noticeable evidence at *p* < 0.001, *p* < 0.01, and *p* < 0.05, respectively.

Springiness and cohesiveness measurements are both associated with the resistance of a product to the second compression (Texture Technologies Corp. 2020). Springiness of lentil couscous ranged from 0.09 to 0.12 and cohesiveness ranged from 0.09 to 0.11. According to Boudouira et al. (2023), springiness and cohesiveness in semolina couscous were observed to decrease with an increase in lentil flour. Similarly, the springiness of our lentil couscous decreased with an exponential increase in *x*_1_ (Table 4), and cohesiveness decreased with an increase in *x*_1_ (Table 4 and Figure 3). Starch is the determining component in gluten‐free pasta products especially if its macromolecular structure can be rearranged to present a similar springy and cohesive texture found in semolina products (Marti and Pagani 2013). Higher levels of *x*_1_ result in lower starch levels in couscous which reduce the concentration of free hydroxyl groups for moisture absorption when rehydrated (Shang et al. 2008). The negative effect of increasing protein levels on springiness and cohesiveness can be counteracted by an increase in extruder screw speed which disrupts starch granules and increases water absorption and swelling (Ek et al. 2021). Formulations 2, 5, 6, 9, and 11 with lower *x*_1_ and higher *x*_2_ (Table 3) notably presented higher springiness and cohesiveness compared to formulations with higher *x*_1_ and lower *x*_2_ (formulations 1 and 8) which are more desirable couscous texture (Giovanelli et al. 2023; Hammami et al. 2022). However, there was no impact of *x*_2_ on cohesiveness and springiness, and higher levels may be needed to observe a noticeable increase (Table 4 and Figure 3).

In addition to cohesiveness, smoothness, and springiness, chewiness and resilience are important characteristics of couscous (Giovanelli et al. 2023; Hammami et al. 2022). According to Boudouira et al. (2023) higher chewiness observed in lentil‐enriched couscous, 23.91N measured at a lower 0.5 mm/s compression speed, had lower scores in a consumer acceptance test relative to field bean‐enriched couscous at 14.57N. The control semolina couscous developed in the study, however, received the highest acceptance scores and presented moderately high chewiness at 20.55N and the highest resilience at 0.17. Among the lentil couscous formulations, formulations 1, 6, 7, 8, 9, and 10 had a lower chewiness while formulations 1, 8, 10, and 11 had lower resilience (Table 3). Chewiness is a product of gumminess and springiness measurements, and resilience denotes the resurgence of a product to its original height after compression (Texture Technologies Corp. 2020).

Studies revealed an increase in hardness and hence chewiness of pasta and couscous is observed with an increase in the addition of pulse flours due to their higher protein content (Boudouira et al. 2023; Bouasla et al. 2018). However, Laleg et al. (2016) explain that a decrease in starch and an increase in legume proteins with high solubility weaken the protein network surrounding starch granules resulting in less chewy and resilient couscous easily solubilized in water. Generally, chewiness and resilience both decreased with an increase in *x*_1_ (Table 4 and Figure 3) indicating an increase in the lentil protein concentrate and a resultant reduction in starch gelatinization which decreases the recovery of lentil couscous to its original height after compression. Chewiness is a textural parameter derived from springiness, and resilience similar to springiness is derived from the compression distance (Texture Technologies Corp. 2020). Therefore, the decrease in springiness observed with an exponential increase in *x*_1_ is reflected in the decrease in chewiness and resilience (Table 4).

Compared to the reference wheat semolina couscous, lentil couscous formulations 7 and 10 were not different in hardness, formulations 1, 5, 10, and 11 were not different in gumminess and formulations 10 and 11 as well did not differ in chewiness. Remarkably, lentil couscous formulations 2–7 and 9–11 were not different in springiness from the wheat semolina couscous. Springiness is an important attribute in couscous and wheat semolina. It is reported to originate from the action of the protein gluten (Benayad et al. 2021). Lentil flour and lentil protein concentrate are gluten‐free (Romano et al. 2021) Therefore, the successful development of “springy” lentil couscous highlights its potential as a noteworthy gluten‐free alternative to wheat semolina couscous. However, the lentil couscous formulations had lower cohesiveness and resilience compared to wheat semolina couscous. Nonstarch polysaccharides such as xanthan gum have been consistently used in gluten‐free formulations to mimic gluten's elasticity and improve texture (Larrosa et al. 2013).

### 3.5 Sensory Evaluations of Lentil Couscous Formulations

The sensory liking of five selected lentil couscous formulations against wheat semolina is shown in Table 5. The overall liking of the selected samples ranged from 5.72 ± 1.62 to 6.37 ± 1.69 (Table 5). Boudouira et al. (2023) reported lower overall liking scores for couscous enriched with lentil flour at 4.85 compared to a score of 7.54 for a control semolina couscous on a nine‐point hedonic scale. Lentil couscous formulation 6, however, presented the highest overall liking of 6.37 ± 1.69 but was found not to be different from formulations 7, 8, and 9 (Table 5). Remarkably, formulations 6 and 7 were found to have noticeably higher liking (*p* < 0.05, Table 5) than wheat semolina which emphasizes the concluding remarks of Benayad et al. (2021), recommending the development of a 100% legume‐based couscous. Conversely, differences in overall liking were not found (*p* > 0.05) between lentil formulations 8, 9, 10, and wheat semolina. This agreed with findings from Demir and Demir (2016) who observed that the overall liking of a 100% wheat semolina couscous did not differ from formulations with lentil flour inclusions.

**TABLE 5:** Sensory liking of selected lentil couscous formulations compared to the reference product, by the participant pool or clusters.

Formulations	Overall			Appearance		
	Pooled	Cluster 1	Cluster 2	Pooled	Cluster 1	Cluster 2
6‐20/80/250	6.37 ± 1.69^a^	4.91 ± 1.19*	7.48 ± 1.04*	6.84 ± 1.43^a^	5.83 ± 1.04*	7.61 ± 1.19*
7‐0/100/250	6.21 ± 1.50^ab^	5.25 ± 1.37*	7.07 ± 1.02*	6.55 ± 1.46^ab^	5.59 ± 1.19*	7.43 ± 1.09*
8‐40/60/250	5.95 ± 1.68^abc^	6.97 ± 1.02*	4.38 ± 1.25*	5.74 ± 1.73^c^	6.20 ± 1.78*	5.02 ± 1.39*
9‐20/80/300	6.10 ± 1.59^abc^	4.47 ± 1.13*	7.00 ± 0.97*	6.50 ± 1.33^ab^	5.66 ± 1.02*	6.97 ± 1.26*
10‐20/80/200	5.72 ± 1.62^c^	4.28 ± 0.89*	6.80 ± 1.12*	6.25 ± 1.50^b^	5.61 ±1.36*	6.74 ± 1.42*
Wheat semolina	5.87 ± 2.01^bc^	6.86 ± 1.49*	3.83 ± 1.25*	6.42 ± 2.09^ab^	7.00 ± 1.93*	5.23 ± 1.91*
Formulations	Flavor			Texture		
	Pooled	Cluster 1	Cluster 2	Pooled	Cluster 1	Cluster 2
6‐20/80/250	6.32 ± 1.75^a^	4.89 ± 1.25*	7.39 ± 1.23*	6.56 ± 1.63^a^	5.30 ± 1.40*	7.51 ± 1.06*
7‐0/100/250	6.20 ± 1.47^ab^	5.33 ± 1.34*	6.98 ± 1.10*	6.33 ± 1.50^ab^	5.18 ± 1.11*	7.38 ± 0.93*
8‐40/60/250	5.89 ± 1.81^ab^	7.06 ± 1.06*	4.07 ± 1.09*	5.98 ± 1.74^bc^	6.74 ± 1.2*	4.81 ± 1.80*
9‐20/80/300	6.15 ± 1.63^ab^	4.79 ± 1.51*	6.90 ± 1.14*	6.10 ± 1.79^abc^	4.61 ± 1.69*	6.93 ± 1.23*
10‐20/80/200	5.85 ± 1.76^ab^	4.22 ± 1.09*	7.08 ±1.00*	5.80 ± 1.82^c^	4.54 ± 1.52*	6.75 ± 1.42*
Wheat semolina	5.83 ± 2.24^b^	7.08 ± 1.44*	3.26 ± 1.09*	5.89 ± 2.26^bc^	6.89 ± 1.76*	3.83 ± 1.72*

^a^ For the same column, values (means ± SD) followed by the same letter are not significantly different based on Fisher's least significant difference test (*α* = 0.05).

^b^ For the same row, values with * indicate significant difference between clusters based on a two‐sample *t*‐test (*α* = 0.05).

The appearance liking of the selected formulations ranged from 5.74 ± 1.73 to 6.84 ± 1.43 (Table 5). Lentil couscous formulation 6 exhibited the highest appearance liking score of 6.84 ± 1.43 but was found not to be different from formulations 7, 9, and wheat semolina. This matched with findings from Demir and Demir (2016) who reported that the inclusion of lentil flour to wheat semolina in the production of couscous did not reduce appearance liking. Lower appearance liking was observed for formulation 8 with higher *x*_1_ levels and formulation 10 with lower *x*_2_ levels (Table 5). Formulation 10 was however found not to be different from formulations 7, 9, and wheat semolina. Formulation 8 was noticeably lower in appearance than all other selected formulations (*p* < 0.05, Table 5). Formulation 8 with 40% lentil protein concentrate was observed to have a comma shape (Figure 1) different from the typical spherical shape of couscous seen in formulations 6, 7, 9, and wheat semolina (Figure 1). The contrasting shape of formulation 8 can be attributed to the reduced starch content which causes a reduction in gelatinization and slight expansion needed to provide the ideal spherical shape of couscous (Giovanelli et al. 2023).

The flavor liking of selected samples exhibited a range from 5.83 ± 2.24 to 6.32 ± 1.75 with formulation 6 presenting the highest score and wheat semolina, the lowest flavor liking score. The higher scoring of formulation 6 compared to wheat semolina can be attributed to the presence of slight beany flavors of pulse‐based nutrition bars after high‐temperature processing which was observed by Szczygiel et al. (2017) to result in higher overall liking. Consequently, the flavor liking of lentil couscous formulations 7–10 was found not to be different from formulation 6.

The texture liking of the selected couscous formulations ranged from 5.80 ± 1.82 to 6.56 ± 1.63 with formulation 6 presenting the highest texture liking of 6.56 ± 1.63 which was found to be different than formulations 8, 10, and the reference wheat semolina. The structure and texture of gluten‐free couscous are reported to be contingent on starch gelatinization and retrogradation (Marti and Pagani 2013). Formulations 8 and 10 have higher levels of lentil protein concentrate and lower screw speeds respectively which limit starch gelatinization and retrogradation on hydration with hot water. The lower texture liking of wheat semolina is attributed to gluten which has been reported by Chemache et al. (2021) to produce stronger cohesive forces that can negatively impact the final texture of couscous when extrusion is used instead of traditional steaming.

Consequently, the overall liking of the presented couscous formulations was found to be strongly positively correlated with flavor liking (0.952) and texture liking (0.980). Texture especially is reported to have a noticeable impact on the culinary quality of couscous (Benayad et al. 2021; Giovanelli et al. 2023; Hammami et al. 2022). Formulations 6 and 7 with higher flavor and textural liking were therefore found to have higher overall liking. In addition, formulations 6 and 7 observably obtained higher liking in all sensory categories, which instigates the possibility of a sensory halo effect.

#### 3.5.1 Check All That Apply

**FIGURE 4:**
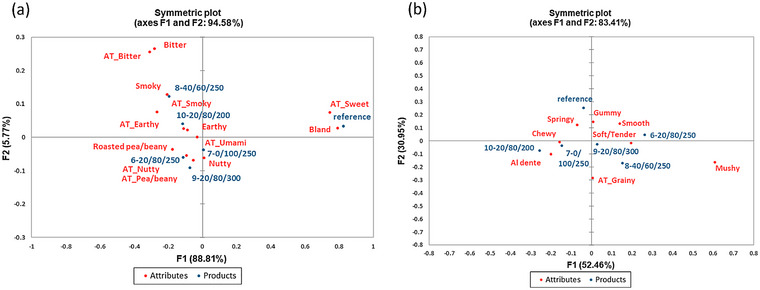
Biplot showing the corresponding CATA flavor (a), and texture (b) attributes of selected lentil couscous formulations.

The results from the CATA test (Figure 4 and Table 6) show the sensory attributes selected by participants out of the 19 presented attributes to describe the flavor and texture of the lentil couscous formulations. Particularly, attributes of nutty, bland, smooth, soft/tender, aftertaste_nutty, and aftertaste_grainy were found to have a noticeable effect (*p* < 0.05) on overall liking (Table 6). Differences in lentil couscous formulations (*p* < 0.05) were only found in smooth, al dente, chewy, and mushy texture attributes (Table 6) out of the 19 presented attributes. Consequently, lentil couscous formulations were located in similar attribute regions (Figure 4), especially for flavor which is either intrinsic in lentils or developed during processing (Vurro et al. 2024). Wheat semolina, however, was in a distant region and featured bland and sweet aftertaste attributes (Figure 4 and Table 6).

**TABLE 6:** Multiple pairwise comparisons of CATA attributes between selected lentil couscous formulations and the wheat semolina.

Attributes^a^	6‐20/80/250	7‐0/100/250	8‐40/60/250	9‐20/80/300	10‐20/80/200	Wheat Semolina	Mean impact_overall liking^b^
Roasted pea/beany	0.598^a^	0.523^a^	0.598^a^	0.570^a^	0.579^a^	0.159^b^	0.099
Nutty	0.561^a^	0.561^a^	0.495^ab^	0.533^a^	0.514^a^	0.318^b^	0.648*
Earthy	0.505^a^	0.430^a^	0.542^a^	0.430^a^	0.458^a^	0.215^b^	0.246
Smoky	0.187^a^	0.150^ab^	0.224^a^	0.112^ab^	0.187^a^	0.047^b^	0.162
Bitter	0.056^ab^	0.065^ab^	0.159^a^	0.093^ab^	0.121^ab^	0.019^b^	−1.214
Bland	0.206^b^	0.308^b^	0.168^b^	0.252^b^	0.252^b^	0.636^a^	−0.775*
Smooth	0.374^a^	0.280^ab^	0.196^b^	0.280^ab^	0.178^b^	0.299^ab^	0.624*
Soft/tender	0.430^a^	0.280^ab^	0.430^a^	0.346^ab^	0.252^b^	0.346^ab^	0.918*
Al dente	0.224^b^	0.364^ab^	0.215^b^	0.318^ab^	0.393^a^	0.215^b^	0.236
Gummy	0.308^a^	0.327^a^	0.243^a^	0.243^a^	0.290^a^	0.402^a^	−0.379
Springy	0.290^b^	0.280^b^	0.299^ab^	0.318^ab^	0.383^ab^	0.467^a^	0.061
Chewy	0.290^b^	0.449^ab^	0.449^ab^	0.449^ab^	0.579^a^	0.523^a^	−0.251
Mushy	0.121^a^	0.019^b^	0.131^a^	0.093^ab^	0.019^b^	0.047^ab^	−1.141
Aftertaste_Pea/bean	0.467^a^	0.383^ab^	0.411^a^	0.505^a^	0.495^a^	0.224^b^	0.244
Aftertaste_Nutty	0.430^a^	0.402^a^	0.430^a^	0.458^a^	0.411^a^	0.178^b^	0.301*
Aftertaste_Earthy	0.374^a^	0.374^a^	0.449^a^	0.374^a^	0.402^a^	0.159^b^	0.160
Aftertaste_Smoky	0.131^ab^	0.121^ab^	0.150^a^	0.093^ab^	0.159^a^	0.019^b^	0.067
Aftertaste_Sweet	0.093^b^	0.112^ab^	0.084^b^	0.075^b^	0.075^b^	0.224^a^	0.433
Aftertaste_Grainy	0.224^ab^	0.234^ab^	0.327^a^	0.234^ab^	0.299^a^	0.112^b^	−0.461*

^a^ For the same row, values (means ± SD) followed by the same letter(s) are not significantly different based on Sheskin's critical difference test (*α* = 0.05).

^b^ Values with * indicate significant mean impact of CATA attributes based on a two‐sample *t*‐test (*α* = 0.05).

**FIGURE 5:**
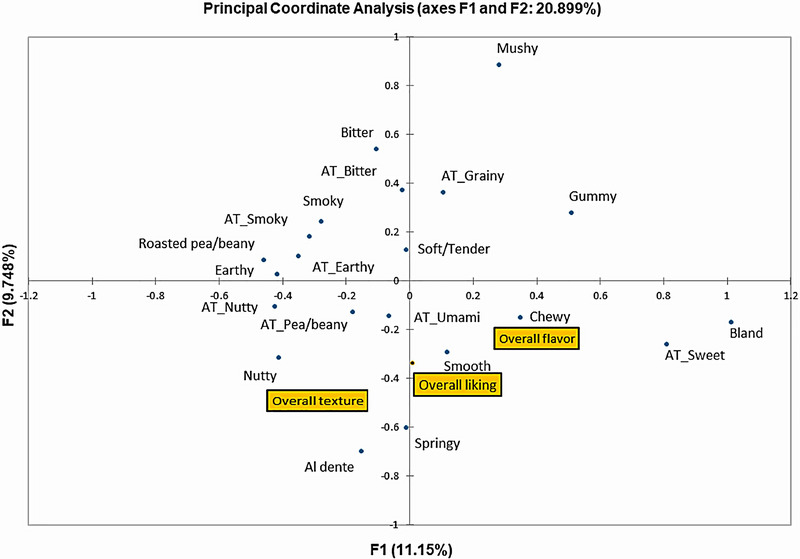
Biplot of the relationship between CATA attributes and overall flavor, overall texture and overall liking of selected lentil couscous formulations.

Generally, lentil couscous formulations 6, 7, and 8, 9 and 10 were found not to differ noticeably (*p* > 0.05) in flavor attributes (Table 6). Wheat semolina, however, was mainly separated from lentil couscous formulations by the reduced presence of roasted pea/beany, nutty, nutty aftertaste, earthy, earthy aftertaste attributes, and the increased presence of bland, and sweet aftertaste attributes (Table 6). Figure 5 further shows that high flavor liking was featured in a region close to umami aftertaste, nutty, nutty aftertaste, roasted pea/beany, and pea/beany aftertaste. In a study by Moussa et al. (2022), the aroma of extruded millet couscous was found to be rated higher than a traditionally prepared millet couscous control. Extrusion particularly has been observed to reduce bitter and off‐flavors in lentils while producing pleasant nutty and roasted flavor from Maillard reactions (Abu‐Ghoush et al. 2015; Vurro et al. 2024). Consequently, nutty, and nutty aftertaste attributes were found to noticeably increase the flavor liking while the bland flavor attribute was found to decrease flavor liking (*p* < 0.05, Table 6).

The texture attributes of cohesive, sticky, smooth, soft/tender, al dente, gummy, springy, chewy, mushy, sticky aftertaste, grainy aftertaste, and creamy aftertaste were selected by participants to describe the selected couscous formulations (Figure 4 and Table 6). Unlike the flavor attributes, the textural attributes were evenly dispersed around couscous formulations (Figure 4). Therefore, clear differences in textural attributes were not found (*p* > 0.05) between lentil couscous formulations and the reference wheat semolina. Figure 5 shows that high texture liking was located near the smooth and soft/tender texture attributes and Table 6 revealed that the smooth and soft/tender texture attributes indeed increase the texture liking of formulations. Gummy and grainy aftertaste attributes were however found to decrease the texture liking of couscous formulations (Table 6). Quality couscous is considered a soft, nonsticky granular product (Abecassis et al. 2012) which explains participants' appreciation for smooth, soft/tender couscous and a decrease in sensory liking for gummy attributes that denote stickiness. None of the samples presented were found close to the region featured by the mushy texture attribute which we assume would also have a negative impact on texture liking.

Overall liking was strongly correlated with flavor liking and texture liking, and this is reflected in associated CATA attributes. Figure 5 therefore shows that the marker for high overall liking was situated near the attributes of texture attributes of smooth, and the flavor attributes of nutty, and nutty aftertaste attributes. Specifically, the attributes of smooth, and soft/tender as well as a nutty and nutty aftertaste, increased overall liking while attributes of bland, gummy, and grainy aftertaste reduced overall liking of presented couscous formulations (Table 6).

### 3.6 Relationship Between Instrumental Texture and Frequency of CATA Texture Attributes

The texture and structure of couscous play a key role in the culinary quality of couscous (Abecassis et al. 2012; Hammami et al. 2022). Pearson correlation analysis was performed between the instrumental texture parameters and the CATA texture attributes of the lentil couscous formulations included in the sensory test to understand the relationship between instrumental and subjective tests of couscous and understand its effect on sensory liking. Instrumental hardness, gumminess, and chewiness texture were found to be positively correlated with the CATA attribute of springy (*p* < 0.05) while cohesiveness and resilience were negatively correlated with a grainy aftertaste (*p* < 0.05). The correlation coefficients between these variables are 0.855, 0.925, and 0.971 between hardness and springy, gumminess and springy, and chewiness and springy, respectively. Instrumental cohesiveness and grainy aftertaste and resilience and grainy aftertaste presented correlation coefficients of −0.862 and −0.939.

Chewiness according to Texture Technologies Corp. (2020) is derived from hardness, cohesiveness, and springiness measurements. The observed higher positive correlation of 0.971 between chewiness and the springy CATA attribute demonstrates that participants were able to detect outstanding qualities of lentil couscous formulations. Remarkably, instrumental cohesiveness and resilience, derived from springiness (Texture Technologies Corp. 2020) are reported to be desirable characteristics of couscous (Hammami et al. 2022) and had a negative correlation of −0.862 and −0.939 respectively with the grainy aftertaste CATA attribute. The grainy aftertaste shown in Table 6 negatively impacts the overall liking of lentil couscous formulations.

### 3.7 Cluster Analysis of the Consumers in Relation to Purchase Indicators of Formulation 6

Agglomerative hierarchical clustering identified two consumer clusters, which were differentiated based on the consumer liking for formulation 6 (Table 5). The consumers in Cluster 1 issued lower overall, appearance, flavor, and texture liking compared to Cluster 2 (Table 5). Cluster 2 with higher overall, appearance, flavor, and texture liking scores had a noticeably higher proportion of women participants (*p* < 0.05) as well as participants who eat lentils once a week compared to the total pool (Table 5). Our findings are consistent with Lemken et al. (2019) who observed that a large proportion of participants who frequently substituted meat for legumes consisted of women. Stoll‐Kleemann and Schmidt (2017) further explain that women are more likely to switch to a plant‐based diet.

The median PI of participants in Cluster 1 was 3 (Table 5), which indicates that participants might or might not buy formulation 6 if it was available in the market. Participants in Cluster 1 also had a mean WTP score of $3.00 ± 0.76 (Table 5) for 10 oz of lentil couscous which is below the $4.18 market price of wheat semolina couscous on the market. Participant in Cluster 2 validated their higher overall sensory liking with a median PI of 4 (Table 5) which indicates that participants are willing to buy lentil couscous formulation 6. The mean WTP scores of $3.36 ± 0.78 for 10 oz of lentil couscous in Cluster 2 was higher than Cluster 1 (Table 5) albeit still lower than the market price for wheat semolina. Kim and Kuo (2022) explain that purchasing value‐added pulse products (VAPPs) is usually intentional since they can be inconvenient for consumers to buy. This aligns with Cluster 2 which issued higher liking scores for being willing to buy the presented lentil couscous compared to Cluster 1 with lower liking scores. However, the low WTP for lentil couscous can be attributed to consumers being likely not to purchase VAPPs if they have not had the chance to become familiar with them (Kim and Kuo 2022).

Therefore, consumer studies on lentils and VAPPs like lentil couscous are needed to determine factors needed to increase consumer engagement and familiarity. Additionally, studies on optimizing the energy required to produce lentil couscous relative to wheat semolina couscous would help justify efforts to utilize diverse ingredients in food production.

## 4 Conclusions

Lentil couscous formulations presented sufficient differences in their bulk density, water absorption and instrumental texture properties, which translated into small differences in sensory liking and CATA attributes perceived by participants. Bulk density, hardness and gumminess texture were surprisingly found to be independent of both factors of weight ratio of lentil protein concentrate to lentil flour and extruder screw speed. Lentil couscous formulations with a maximum lentil protein concentrate level of 20% and a minimum screw speed of 250 rpm presented sensory liking above 6.0 on a nine‐point hedonic scale. Specifically, sensory flavor attributes of nutty and a nutty aftertaste and texture attributes of smooth and soft/tender were found to be desired by participants in the consumer acceptance test. Consequently, clusters obtained from grouping the sensory liking of participants into low and high revealed that participants who issued higher sensory scores had more women compared to the total pool of participants. The high‐scoring cluster was also willing to buy the presented sensory samples but for $3.37 for 10 oz of lentil couscous.

The successful development of nutty, smooth, and soft couscous from a 100% lentil base highlights the feasibility of lentils as a viable replacement for wheat in couscous and other pasta‐based products. The created lentil couscous provides a gluten‐free pasta‐based product with increased protein and fiber, a pleasant nutty flavor, smooth and soft texture, promising to increase US appreciation for whole lentil products.

## Author Contributions

**Edwin Allan**: conceptualization, investigation, writing–original draft, methodology, validation, visualization, writing–review and editing, software, formal analysis, project administration, data curation. **Dilpreet Bajwa**: supervision, writing–review and editing. **Girish M. Ganjyal**: methodology, writing–review and editing, supervision. **Chidimma Ifeh**: methodology, writing–review and editing. **Ravi Pirati**: methodology, writing–review and editing, investigation. **Wan‐Yuan Kuo**: conceptualization, investigation, funding acquisition, methodology, writing–review and editing, project administration, supervision, resources.

## Conflicts of Interest

The authors declare no conflicts of interest.

## Supporting information




**Supplementary Table**: jfds70356‐supp‐0001‐Tables.docx
