# Supplementary material for: Exploring the Potential of Flour and Protein Concentrate From Richlea Lentils in the Development of Couscous With a Pilot‐Scale Twin‐Screw Extruder
Source: J Food Sci. 2025 Jul 14;90(7):e70356. doi: 10.1111/1750-3841.70356 (PMC12260130; doi:10.1111/1750-3841.70356)
Supplement: Supplementary file 1 — Supplementary Table: jfds70356‐supp‐0001‐Tables.docx [file JFDS-90-0-s001.docx]

Table 7: Demographic breakdown of the clusters based on sensory scores.

| Demographic groups | | Total Pool | Cluster 1 | Cluster 2 |
| --- | --- | --- | --- | --- |
| Gender | Man | 38 (35.51%) | 21 (45.65%) | 17 (27.87%) |
|  | Woman | 67 (62.61%) | **24 (52.17%)*** | **43 (70.49%)*** |
|  | Prefer not to answer | 2 (0.02%) | 1 (2.17%) | 1 (1.64%) |
| Age | 18-34 | 78 (72.90%) | 33 (71.73%) | 45 (77.77%) |
|  | 35-44 | 13 (12.15%) | 6 (13.04%) | 7 (11.48%) |
|  | Above 44 | 16 (14.95%) | 7 (15.22%) | 9 (14.76%) |
| Ethnicity | White | 87 (81.31%) | 38 (82.61%) | 49 (80.33%) |
|  | Non-white | 19 (17.76%) | 8 (17.38%) | 11 (18.04%) |
|  | Prefer not to answer | 1 (0.01%) | 0 (0%) | 1 (1.64%) |
| Eat lentils | Never | 5 (4.67%) | 3 (6.52%) | 2 (3.28%) |
|  | Occasionally | 63 (58.88%) | 30 (65.21%) | 33 (54.10%) |
|  | Once a week | 24 (22.43%) | 7 (15.22%) | 17 (27.87%) |
|  | 2-3 times a week | 10 9.35%) | 2 (4.35%) | 8 (13.12%) |
|  | 4-6 times a week | 5 (4.67%) | 4 (8.70%) | 1 (1.64%) |
| Eat couscous | Never | 27 (25.23%) | 14 (30.44%) | 13 (21.31%) |
|  | Occasionally | 68 (63.55%) | 26 (56.52%) | 42 (68.85%) |
|  | Once a week | 6 (5.61%) | 4 (8.70%) | 2 (3.28%) |
|  | 2-3 times a week | 2 (1.87%) | 0 (0%) | 2 (3.28%) |
|  | 4-6 times a week | 4 (3.74%) | 2 (4.34%) | 2 (3.27%) |

The results are displayed as the number of individuals and the corresponding percentage relative to the total in the cluster. The percentage followed by an * indicates a significant difference from the pooled percentage based on Fisher's exact test (α = 0.05).
